# Improving safe post-abortion care practices: A study on interventions implemented by Ipas Pakistan

**DOI:** 10.3389/fpubh.2023.1004381

**Published:** 2023-03-06

**Authors:** Amna Arshad, Hina Aziz, Ghulam Shabbir, Sagun Shakya, Zarnab Munir

**Affiliations:** ^1^Ipas, Islamabad, Pakistan; ^2^Ipas, Chapel Hill, NC, United States

**Keywords:** post-abortion care, manual vacuum aspiration, medical abortion, misoprostol, post-abortion family planning

## Abstract

An estimated 50 million induced abortions occur in developing countries annually, and an estimated 7 million women are treated for complications associated with unsafe abortions. According to a 2012 estimate, 15 per 1,000 women aged 15–49 years seek treatment for abortion-related health complications in both private and public sectors. A high unmet need for family planning in Pakistan and a low percentage of women adopting a contraceptive method in the post-partum period led to unwanted pregnancy becoming one of the reasons for unsafe abortion. Post-abortion care (PAC) is an integrated service delivery model that includes both maternal health and family planning interventions. The study aims to examine improvement in abortion-related practices through the implementation of the PAC model at all tiers of public health service delivery systems in the two most populous provinces—Punjab, Sindh, Khyber Pakhtunkhwa, and Islamabad Capital Territory (ICT) region—of Pakistan. The improvement model comprises clinical training of healthcare providers, community engagement, and counseling of community women on safe post-abortion practices. It was a descriptive study utilizing data of 27,616 PAC clients recorded and reported by the service providers on the logbooks from 104 selected public health facilities from March 2018 to December 2021 in ICT, Punjab, Sindh, and Khyber Pakhtunkhwa provinces of Pakistan. Women who received PAC services were older than 25 years, 22,652 (82%), with a mean age of 29 years. Most of these women were in their first trimester, 26,110 (95%), and the majority diagnosed with PAC (incomplete, missed, or threatened abortion), 26,838 (97%). The majority of women, 25,324 (92%), received safe methods for post-abortion care that included the use of misoprostol, 15,804 (58%), and manual vacuum aspiration, 8,898 (32%). In total, 17,105 (72%) of women opted for a contraceptive method that included long-acting reversible contraceptives, 2,313 (10%); short-term excluding condoms, 3,436 (27%); and condoms, 8,113 (34%). The key predictors identified for uptake of the post-abortion family planning method indicated that women more than 25 years of age, in the early second trimester, and who were counseled on post-abortion family planning were more likely to adopt the contraceptive method than others. Increased access to post-abortion care and family planning could potentially reduce the incidence of unsafe abortion, unintended pregnancies, and associated maternal mortality. The experience of Pakistan suggests that the integrated post-abortion care service delivery model can be effectively implemented across the public health systems.

## 1. Introduction

An estimated 50 million induced abortions occur in developing countries annually, many taking place outside of health facilities (1). A large proportion, however, is carried out in health facilities in the public, private, and non-governmental organization (NGO) sectors, and an estimated 7 million women are treated for complications from unsafe abortions performed every year (2).

Unsafe abortion performed by sharp curettage continues to be a common cause of maternal deaths in Pakistan, and more than 85% of the women living in rural areas do not have access to skilled attendants contributing to the burden of maternal mortality and morbidity. Despite changes in the legislation implemented in 1997 allowing pregnancy termination not only when required to save the woman's life but also in the early stages of pregnancy when organs are not formed to provide essential treatment (3). However, no additional definition of this term was established, leaving its understanding open to healthcare providers and the person seeking the desired services. The WHO and the International Federation of Gynecology and Obstetrics (FIGO) state that manual vacuum aspiration or medication regimens should replace sharp curettage. Sharp curettage performed alone or with a combination of vacuum aspiration was significantly associated with complications than vacuum aspiration without curettage (4, 5). Though the community and most of the policymakers and healthcare providers remain unaware of this change in legislation, no public sector hospital executes pregnancy terminations for any indication other than therapeutic abortions for severe fetal anomalies and to save the life of the mother. This is also due to high abortion-related stigma, providers' bias, myths and misconceptions, and the substantial emergency workload of the public sector hospitals. Many women who experience an unintended pregnancy due to the low prevalence of contraceptive use resort to induced abortion. Due to the lack of proper equipment and availability of supplies and biases attached to abortion and post-abortion care in public sector health facilities, most women seek induced abortions from the private sector by informal and/or untrained healthcare providers such as traditional birth attendants and Dais (6). A study carried out by the Population Council in 2012 overall estimated that 15 per 1,000 women aged 15–49 years seek treatment for abortion-related health complications in both private and public sectors. Though the average caseload at public health facilities declined during the last decade, however, it increased at public teaching hospitals and rural health centers (7).

Post-abortion care (PAC) is an integrated service delivery model that has five elements including (1) treatment, (2) counseling, (3) contraceptive and family planning services, (4) reproductive and other health services, and (5) community and service provider partnerships. This model includes both uterine evacuations (UE) through safe methods of manual vacuum aspiration (MVA) and medical use of misoprostol and post-abortion family planning (PAFP) interventions, which are both curative and preventative. As unplanned pregnancy plays a vital role in defining the unmet need for family planning, to the extent that the termination of an unwanted pregnancy is a motivating factor for induced abortion (8).

Pakistan has one of the highest percentages of married women with unmet needs for family planning (17%) (9). In Pakistan, getting direct information from women about abortion and post-abortion complications is tough because of the stigma and hesitancy attached to the answers (7).

Aimed to improve the sexual and reproductive health of women and girls, Ipas, an international non-governmental organization (INGO) provided women-centered post-abortion care (WC-PAC) clinical training to doctors and mid-level providers followed by mentoring, onsite support, and seed supply of essential PAC (post-abortion care) technologies. To strengthen the referral mechanism and linkages, post-abortion care information was provided to community women through community engagement interventions and intermediaries. Concurrently, policymakers and stakeholders were onboarded and sensitized on safe post-abortion technologies and their importance in reducing the maternal mortality associated with unsafe practices. However, this article explicitly explains the Ipas Pakistan PAC model implemented at public care-level health facilities across the three provinces Punjab, Sindh, Khyber Pakhtunkhwa, and the ICT region of Pakistan. The model integrates WHO-recommended safe methods and counseling procedures and service delivery data collected *via* logbooks used as a data collection tool after providing the desired services to the community women at the public health facilities, to examine improvement in safe post-abortion practices across Pakistan.

## 2. Materials and methods

### 2.1. Study design and sample

It was a descriptive study utilizing data from 27,616 PAC clients who were treated for post-abortion care at tertiary to secondary to primary care-level healthcare facilities from 15 October 2018 to 31 March 2021.

### 2.2. Coverage and intervention model

The interventions were initially implemented in Punjab (93), Islamabad Capital Territory/Rawalpindi ICT/RWP division (7), and were later phased out to other health facilities of Sindh (11) and Khyber Pakhtunkhwa (2). The facilities included all levels of primary, secondary, and tertiary healthcare centers including 26 basic health units (BHU), 45 rural health centers (RHC), eight district headquarter hospitals (DHQH), 27 tehsil headquarter hospitals (THQH), and seven tertiary care teaching hospitals.

The interventions included the selection of appropriate facilities and providers including both obstetrics and gynecology consultants/doctors and mid-level providers, equipment upgrade for service provision, clinical training of providers, values clarification, and attitude transformation (VCAT), and offering follow-up clinical and programmatic support to providers and facilities, respectively, as much and as often as needed. A standard procedure was followed for selecting the facilities that included site baseline assessment, provider baseline assessment based on the experience, availability to attend 5 days of clinical training, willingness to pursue clinical practice in the gynae department, commodity supply, and evaluation of existing data recording mechanism on any type of abortion methods provided at the facility were conducted at the time of baseline assessment. It was found that proper data recording for abortion clients mentioning diagnosis and method performed was absent in the majority of the facilities. Following selection, the facilities were provided essential equipment upgrades, seed supply of PAC-FP commodities, and support to provide standard care to community women related to PAC and PAFP, with proper counseling and commodities provided at the same location.

An important component of the intervention was clinical skilled competency-based women-centered post-abortion care (WC-PAC) training that included values clarification and attitude transformation (VCAT) sessions, safe methods including medical use of misoprostol and MVA, training on long-acting reversible contraceptive methods (implant and IUCD insertion), and record-keeping. At the time of training, providers were evaluated through pre- and post-tests related to clinical knowledge for PAC procedures and related care performed correctly as determined by a clinical trainer utilizing competency checklists. Logbooks were also provided for service delivery data reporting of all the PAC and PAFP clients after training. A total of 352 providers were trained including 159 doctors/specialists, 193 mid-level providers including 105 lady health visitors (LHVs), and 88 registered nurses and midwives (RN/RM). In total, 47% (165) of the providers were trained from primary care-level facilities, 35% (123) from secondary care-level facilities, and 18% (64) from tertiary care-level facilities.

### 2.3. Data recording and management

Healthcare providers were trained to record service delivery clinical client-level data at the facility on the logbooks on regular basis. The logbook data were periodically collected, monitored, and analyzed by the Ipas team in terms of caseload generated by each facility and shift from unsafe to safe practices by the providers. The variables on which clinical data were recorded included diagnosis (induced abortion or PAC), the technology used for uterine evacuation, MVA, electric vacuum aspiration (EVA), dilation and evacuation (D&E), sharp curettage (D&C), and medical abortion *via* misoprostol, gestation age determined through last mensuration period (LMP), information on parity and gravida, pain management, reporting on complications, serious adverse events, counseling on contraception, and client uptake of post-abortion contraception method. Each trained provider and facility have been assigned an identification code to record each case they attended, allowing the performance to be monitored.

### 2.4. Data analysis

Analysis was performed using STATA version 14. Descriptive univariate analysis was conducted of all dependent and independent variables. Bivariate relationships were examined between the Ipas-trained and non-Ipas-trained providers. Finally, multivariate logistic regression was performed for the uptake of the post-abortion family planning method after utilizing each outcome in the models.

## 3. Results

**Figure 1:**
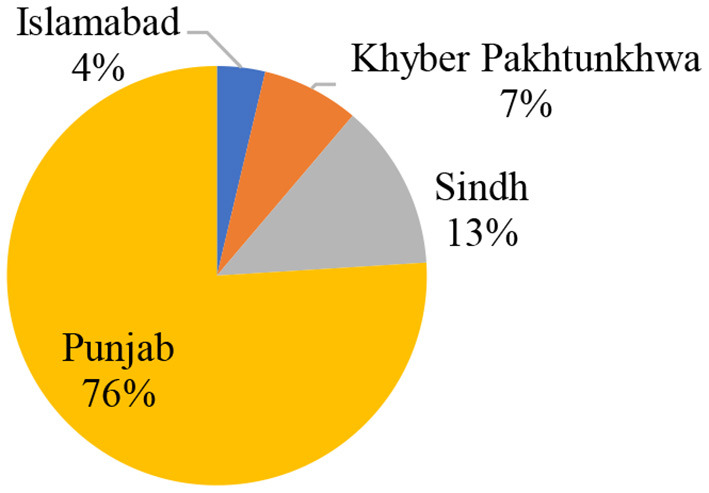
Number of PAC clients reported from the provinces.

This analysis is based on 27,616 PAC clients provided with post-abortion care services at 104 facilities from 15 October 2018 to 31 March 2021. A higher number of cases were reported from Punjab, 20,985 (76%), followed by Sindh, 3,539 (13%), Khyber Pakhtunkhwa, 2,068 (7%), and Federal Capital Territory (Islamabad), 1,024 (4%) (Figure 1).

A majority of PAC clients were reported from the secondary care-level facilities, 12,272 (45%), followed by primary care-level facilities, 11,081 (40%), and from tertiary care-level facilities, 4,263 (15%) (Table 1). Healthcare providers were categorized into two main groups, i.e., those who were trained by Ipas including doctors/specialists and mid-level providers (Ipas-trained providers). The second category includes those healthcare providers who were not trained by Ipas but were providing services within the same facilities (non-Ipas-trained providers). The data indicate that most of the PAC clients, 13,004 (47%), were managed by mid-level providers which include LHVs, 7,857 (28%), and RN/RM, 5,147 (19%). This was followed by doctors, 10,038(36%), and non-Ipas-trained providers, 4,574 (17%). The caseload was found to be more at the secondary care level, whereas 35% of Ipas-trained providers were available in these facilities.

**Table 1:** Number of PAC clients reported from different types of facility (*n* = 104) and healthcare provider (*n* = 309).

**Type of health facility**	**No. of clients**	**Percentage**
	**(*n =* 27,616)**	**(%)**
**Primary care level**	11,081	40
BHU	390	1
RHC	10,772	39
**Secondary care level**	12,272	45
DHQ	3,490	13
THQ	8,782	32
Teaching/tertiary care level	4,263	15
**Health care providers**		
Doctors	10,038	36
Mid-level provider	13,004	47
Non-Ipas trained	4,574	17

A majority of the women utilizing PAC services were older than 25 years, 22,652 (82%), with a mean age of 29 years. Most of the women were in their first trimester, 26,110 (95%), and most were diagnosed with incomplete abortion, 20,931 (93%). The majority, 23,014 (83%), of clients reported self-referral, while only, 4,483(16%), reported referrals from a lady health worker (LHW) who was trained on social behavioral change (SBC) tools under community engagement intervention to strengthen community linkages and referrals.

Post-abortion care (PAC) methods were classified based on the approaches labeled as safe and unsafe methods. Uterine evacuation to remove the retained pregnancy tissue is often a lifesaving component of PAC and includes the use of misoprostol, MVA, EVA, and D&E, whereas the unsafe method includes sharp curettage (D&C). The data indicate that the majority of women, 21,893 (95%), received safe methods for post-abortion care compared to 1,149 (5%) who underwent unsafe methods. Women also received counseling on post-abortion family planning methods, 26,255 (95%), and approximately 72% opted for a method that included LARCS (10%) including implant (2%), IUCD (8%), short-term excluding condoms (27%), condoms (34%), and permanent (1%) (Table 2).

**Table 2:** Characteristics of women receiving PAC services at the health facility.

**Variable**	**Categories**	***n***	**%**
Age of the women	19–24	4,936	18
	>25	22,652	82
	Missing	28	0
	Total	27,616	100
Gestational age	First Trimester (0 to 13 weeks)	26,110	94.5
	Second Trimester (>13 weeks)	1,258	4.5
	Other/missing	248	1%
	Total	27,616	100
Referral to the facility	Self	22,124	80
	LHW	4,265	15
	Others (private clinic, pharmacy, community midwife, traditional birth attendant)	1,227	5
	Total	27,616	100
Type of abortion	Postabortion care (Incomplete/missed/threatened)	26,838	97
	Others (spontaneous, retained products of conception-RPOCs)	778	3
	Total	27,616	100
Post-abortion care method used for postabortion care	Safe method	25,324	92
	Misoprostol alone	15,804	58
	MVA	8,898	32
	Dilation and Evacuation	2,78	1
	EVA	310	1
	Unsafe method (Sharp curettage)	1,784	6
	Others (sponge-holding forceps)	506	2
	Total	26,838	100
Counseling for post-abortion family planning uptake	Yes	26,255	95
	No	1,324	5
	Missing information	37	0
	Total	27,616	100
PAFP method uptake	Condoms	9,384	34
	Short term (Excluding condoms)	7,460	27
	LARCs (Implant/IUCD)	2,653	10
	Permanent (tubal ligation)	243	1
	None/Missing	7,876	28
	Total	27,616	100

Inferential analysis was conducted to compare Ipas-trained providers and non-Ipas-trained providers regarding PAC services offered by them (Table 3). This difference was found to be significant with a *p*-value < 0.05. As the tertiary care-level health facilities have providers who went through fellowship training, Fellow of College of Physicians and Surgeons (FCPS, Pakistan), and other in-service trainings, most of them are senior-level staff. The intervention focus was to train providers from primary care- and secondary care-level facilities to fill the gap and to manage the caseload at the primary care- and secondary care-level facilities rather than increasing the unnecessary referrals to the tertiary care-level facilities. Similarly, a significant difference was found in providing PAC through unsafe methods among Ipas-trained and non-Ipas-trained providers. Unsafe method (D&C) provision was low among Ipas-trained providers of only 5%, as compared with non-Ipas-trained providers performed 25% unsafe method (D&C).

**Table 3:** Inferential analysis between Ipas-trained and non-Ipas-trained providers.

**Variables**		**Ipas trained provider**		**Non-Ipas trained provider**		***P*-value**
		***n***	**%**	***n***	**%**	
Health facility	Primary care level	10,030	44	1,051	23	0.000^*^
	Secondary care level	10,010	43	2,262	49	
	Tertiary care level	3,002	13	1,261	28	
PAC method used	Safe methods	21,893	95	3,431	75	0.000^*^
	Unsafe methods	1,149	5	1,143	25	
Counseling provided	Provided	22,082	96	4,174	91	0.000^*^
	Not provided	962	4	403	9	
PAFP method uptake	Condoms	7,537	33	1,847	40	0.000^*^
	Short term(Excluding condoms)	6,563	28	897	20	
	LARCs	2,328	10	325	7	
	Permanent (Bilateral tubal ligation)	189	1	54	1	
	No method	6,425	28	1,451	32	

* The chi-square statistic is significant at the 0.05 level.

Safe method provision was significantly high about 95% through Ipas-trained providers with a *p*-value < 0.05. Regarding post-abortion family planning counseling, 96% of the women were provided counseling by Ipas-trained providers when compared to 91% by non-Ipas-trained providers. Condoms were found to be the most popular methods adopted followed by short-term methods, LARCS, and bilateral tubal ligation (BTL) (Table 3). The PAFP method uptake was comparatively higher among women provided service by Ipas-trained providers (72%) vs non-Ipas-trained providers (68%).

The model indicates that women more than 25 years of age were 1.34 times more likely to adopt a post-abortion family planning method than the women in the younger age group. Similarly, women visiting the facility for PAC in the early second trimester are 0.66 times more likely to uptake a PAFP method than those women who utilize PAC services in the first trimester. In addition, those who were counseled and received information on post-abortion family planning by the healthcare provider were 5.53 times more likely to adopt the method compared with those who were not counseled (Table 3). However, no significant difference was found when compared with Ipas-trained or non-Ipas-trained providers. Furthermore, multivariate logistic regression analysis was conducted to identify the predictors for uptake of post-abortion family planning among women using PAC services with the approach of parsimonious model building and screening criteria of a *p*-value < 0.20 at the univariate level and a *p*-value < 0.05 at the multivariate level. The model preparation was subjected to the Hosmer and Lemeshow goodness-of-fit test. The final derived multivariate model was found to be a good fit (*p*-value < 0.90) (Table 4).

**Table 4:** Logistic regression model for the uptake of post-abortion contraception.

**Characteristic**	**β**	**OR - Adj**	**95% CI of OR**	***P*-value**
Age >25 years	0.296	1.344	1.25–1.44	0
Second trimester	0.407	0.665	0.58–0.75	0
Counseling provided	1.71	5.533	4.47–6.84	0
Ipas trained provider	0.047	1.048	0.97–1.13	0.232^*^

* Hosmer and Lemeshow goodness-of-fit test p-value < 0.9.

## 4. Discussion

Our findings overall indicate that the set of interventions implemented by Ipas has effectively supported improving the post-abortion care and post-abortion family planning uptake in selected districts across the country. Ipas supports the public health facilities in terms of providing clinical training to the providers, mentoring and networking sessions, VCATs, and timely refilling of the commodities such as misoprostol, MVA, implants, and IUCDs showed safe method provision throughout the intervention period. To increase access to safe post-abortion services, training and VCAT activities were conducted to address the provider's biases, myths, misconceptions, introduction to abortion law, provisions therein, and accordingly its correct interpretation. The caseload from October 2018 onward reported from the facilities on the logbooks and regular follow-up meetings with the district health authorities, stakeholders, and facility management indicates that there is a shift toward safe method provision.

These findings are in keeping with the evidence that has led to global recognition of PAC as a high-impact practice (10). The caseload was found to be more at the secondary care-level facilities, possibly due to the greater number of trained providers (35%) available in these facilities. Prior studies indicate that the availability of trained providers in a facility influences post-abortion care services may result in a greater number of women visiting the facility (11, 12). The majority of women (92%) visiting the facilities received a safe method for post-abortion care. This includes the use of misoprostol (58%) and the use of MVA (32%). According to the WHO, safe post-abortion practices with misoprostol and MVA are considered safe methods for uterine evacuation (13). A significant difference was found in providing PAC through unsafe methods among Ipas-trained and non-Ipas-trained providers. Non-Ipas-trained providers provided PAC services through unsafe methods to 25% of the women, whereas only 5% of unsafe methods were performed by Ipas-trained providers. However, there is evidence from many countries including Pakistan that healthcare providers that are not trained to use modern and safe methods subsequently continue to use procedures like dilatation and curettage (D&C) (7). They often lack competency in providing PAC, which is a further barrier to accessing quality services (14), though women preferred medical over surgical treatment for incomplete abortions because of its convenience, limited complications, no requirement for anesthesia, and less pain (15). Therefore, involving a wider range of trained health workers in this intervention is an important public health strategy to address the shortages of trained providers and improve accessibility and availability of safe post-abortion services for women (16).

Our findings further indicate that before the implementation of these interventions, the baseline assessment shows minimal data reporting abortion clients and since October 2018 the reporting practice has significantly improved. Although a decline can be seen from February 2020 possibly owing to the COVID-19 pandemic which was at its peak during these months in Pakistan. Globally, the COVID-19 crisis has created unparalleled limitations on healthcare systems, including inpatient and outpatient, emergency care, and surgical services. Nevertheless, women will always need access to safe post-abortion services. The unfolding COVID-19 crisis is limiting access to contraception and safe post-abortion services, with the poorest and marginalized women and girls being the worst affected. Interruptions in supply chains are leading to a shortage of medications and contraceptives. Economic insecurity is restricting people's ability to pay for services. Quarantines and travel bans are making physical access to safe services increasingly challenging (17). Therefore, we must look at other ways of mitigating these effects, such as reduced in-person clinical visits, expanded availability *via* telemedicine, and task shifting by training and supporting more community-based workers (LHW/LHS) to improve access to safe methods such as medical abortion and abortion self-care during COVID-19 peak times.

In addition, our findings indicate that 95% of the women received counseling on post-abortion family planning methods and ~72% opted for a method. Literature indicates that the trained providers can affect contraceptive uptake as well as method mix by appropriate counseling on a range of methods. Multiple studies to evaluate supportive supervision demonstrate its highest impact on counseling and communications techniques, however, these outcomes are also influenced by the consistent availability of short- and long-acting commodities in the facility and women's preferences for specific methods (11). All contraceptive methods are safe to use after PAC treatment, whether it is performed medically, *via* vacuum aspiration, or surgically to remove residual products of conception. When treated medically, a PAC client may start using hormonal methods including oral contraceptives, injectables, and implants immediately after the onset of treatment, and an intrauterine contraceptive device (IUCD) may be placed when it is certain that the uterus is empty. After vacuum aspiration, all methods, including IUCDs and implants, may be started immediately following a first- or second-trimester procedure (18). However, in our study, condoms were found to be the most popular method adopted by women (34%). This finding is similar to our National demographic health survey data which indicate that the modern contraceptive method uptake is 25% and the most common method (9%) used by currently married women in Pakistan is male condoms (19). This finding is consistent with other research studies which also indicate that uptake of long-term post-abortion family planning continuation and uptake remains thin and inconclusive (18, 20).

Our regression model identified the key predictors for the uptake of post-abortion family planning among the women using PAC services. The model indicates that women more than 25 years of age and in their early second trimester and who received counseling on post-abortion family planning by the healthcare provider were significantly more likely to adopt the method compared to those who were younger, in the first trimester and who were not counseled. However, no significant difference was found when compared with Ipas-trained or non-Ipas-trained providers.

The limitations of our analysis and findings take in the fact that baseline data were not available as a proper data recording mechanism was missing for abortion clients on the facility registers/DHIS along with the availability of limited PAC methods, particularly MVA instruments were not available at most of the facilities. Furthermore, data were recorded by the healthcare providers themselves and therefore had the potential for underreporting, especially given the stigma associated with abortion care, especially in Pakistan. Third, due to COVID-19 restrictions in the latter part of the study, physical verification of the data was not possible and data reporting mainly relied on virtual methods. Our logbook data were also limited, and as a result, we were not able to cover further information such as the number of living children, reasons for abortion, and prior use of contraceptives that may have added benefits while analyzing the data. Due to the limitation of the data, these providers could not be categorized further. It was found that Ipas-trained providers provided services to the majority of clients at primary (41%) and secondary care levels (45%), as compared to non-Ipas-trained providers catering to more clients at the secondary care (50%) and tertiary levels (27%).

## 5. Conclusion

Expanding access to PAC and PAFP could potentially reduce the incidence of unsafe abortion, unintended pregnancies, and associated maternal mortality. The experience of improving PAC services in Pakistan suggests that the integrated PAC service delivery model can be effectively implemented and sustained within the public health system and government to include procurement of commodities including MVA, appropriately maintaining the supply chain, training, and logistics pertinent to abortion methods as well as seed supply of contraceptive commodities. Interaction of PAC data collection as a part of regular recording and reporting would be another best practice based upon which facilities can efficiently manage service provision and supply of commodities. Effective management or care for abortion can in turn increase post-abortion family planning use and can prevent future unwanted pregnancies. Thus, there are opportunities to apply the experience reported in this study to the effect of increasing safe post-abortion care services to women at the national level. Further implemented systematically and at a larger scale, this may reinforce systems and make gains against preventable maternal morbidity and mortality.

## Data availability statement

The raw data supporting the conclusions of this article will be made available by the authors, without undue reservation.

## Author contributions

HA and AA: conceptualization and writing of original draft preparation. AA: methodology, data curation, formal analysis, and investigation. GS, SS, and ZM: validation and review. GS: resource availability. ZM: project administration. SS and GS: writing review and editing and visualization. All authors have read and agree to the published version of the manuscript.
